# Angiotensin II type 1 receptor agonistic autoantibody blockade improves postpartum hypertension and cardiac mitochondrial function in rat model of preeclampsia

**DOI:** 10.1186/s13293-021-00396-x

**Published:** 2021-11-02

**Authors:** George W. Booz, Daniel Kennedy, Michael Bowling, Taprieka Robinson, Daniel Azubuike, Brandon Fisher, Karen Brooks, Pooja Chinthakuntla, Ngoc H. Hoang, Jonathan P. Hosler, Mark W. Cunningham

**Affiliations:** 1 Department of Pharmacology and Toxicology, University of Mississippi Medical Center, Jackson, MS USA; 2 Department of Cell and Molecular Biology, University of Mississippi Medical Center, Jackson, MS USA; 3 Department of Physiology and Anatomy, University of North Texas Health Science Center, 3500 Camp Bowie Boulevard, Fort Worth, TX 76107 USA

**Keywords:** Postpartum, Hypertension, Cardiac mitochondrial function, Cardiovascular disease, Preeclampsia

## Abstract

Women with preeclampsia (PE) have a greater risk of developing hypertension, cardiovascular disease (CVD), and renal disease later in life. Angiotensin II type I receptor agonistic autoantibodies (AT1-AAs) are elevated in women with PE during pregnancy and up to 2-year postpartum (PP), and in the reduced uterine perfusion pressure (RUPP) rat model of PE. Blockade of AT1-AA with a specific 7 amino acid peptide binding sequence (‘n7AAc’) improves pathophysiology observed in RUPP rats; however, the long-term effects of AT1-AA inhibition in PP is unknown. Pregnant Sprague Dawley rats were divided into three groups: normal pregnant (NP) (*n* = 16), RUPP (*n* = 15), and RUPP + ‘n7AAc’ (*n* = 16). Gestational day 14, RUPP surgery was performed and ‘n7AAc’ (144 μg/day) administered via osmotic minipump. At 10-week PP, mean arterial pressure (MAP), renal glomerular filtration rate (GFR) and cardiac functions, and cardiac mitochondria function were assessed. MAP was elevated PP in RUPP vs. NP (126 ± 4 vs. 116 ± 3 mmHg, *p* < 0.05), but was normalized in in RUPP + ‘n7AAc’ (109 ± 3 mmHg) vs. RUPP (*p* < 0.05). PP heart size was reduced by RUPP + ’n7AAc’ vs. RUPP rats (*p* < 0.05). Complex IV protein abundance and enzymatic activity, along with glutamate/malate-driven respiration (complexes I, III, and IV), were reduced in the heart of RUPP vs. NP rats which was prevented with ‘n7AAc’. AT1-AA inhibition during pregnancy not only improves blood pressure and pathophysiology of PE in rats during pregnancy, but also long-term changes in blood pressure, cardiac hypertrophy, and cardiac mitochondrial function PP.

**Supplementary Information:**

The online version contains supplementary material available at 10.1186/s13293-021-00396-x.

## Background

Women with hypertensive pregnancies, such as preeclampsia, are at a greater risk of developing hypertension, cardiovascular disease (CVD), and renal disease later in life [1]. Preeclampsia is a disease that is characterized as new onset hypertension usually occurring in the second to third trimester of pregnancy. Preeclampsia is associated with placental ischemia, oxidative stress, inflammation, endothelial dysfunction, angiotensin II type 1 receptor agonistic autoantibody (AT1-AA) production, and mitochondrial dysfunction [1–7]. Studies from our group examining a placental ischemic animal model of preeclampsia have shown that mitochondrial dysfunction is present in the kidneys and placenta during pregnancy [8–10]. The heart has a high metabolic demand and is rich in mitochondria with some 35% of the heart’s volume being composed of mitochondria [11, 12]. As of today no studies have examined cardiac mitochondrial dysfunction during pregnancy and beyond.

Several clinical studies have suggested that the greater the severity of preeclampsia, the higher the mother’s risk of death and CVD [13–17]. Furthermore, there is robust link between preeclampsia and end-stage renal disease, proteinuria, and a decrease in renal function in preeclamptic women postpartum [1, 18–28]. Women with preeclampsia go on to develop hypertension ~ 10 years earlier than those having a normal pregnancy, putting them at a greater risk of CVD and renal disease [15, 29].

AT1-AAs are elevated during pregnancy and postpartum in preeclamptic women and during pregnancy in the reduced uterine perfusion pressure (RUPP) preclinical rat model of preeclampsia [1, 4, 8, 9, 30–36]. The exact role of AT1-AAs in preeclampsia during pregnancy has been explored in previous studies by our group and others, showing that AT1-AAs increase blood pressure, synergistically enhance angiotensin II (ANG II) AT1-induced renal vascular resistance, proteinuria, and decrease GFR [30, 37, 38]. AT1-AA inhibition, by our inhibitory peptide, which consists of the seven amino acid sequence specific to the epitope binding site of AT1-AA to the AT1 receptor (‘n7AAc’), administered to RUPP rats during pregnancy improves blood pressure and many of the other pathophysiological factors associated with preeclampsia [39]. AT1-AA inhibition in the RUPP preclinical rat model of preeclampsia improves placental and renal mitochondrial function and oxidative stress during pregnancy [8–10].

Although we have seen major improvements in the pathophysiology of preeclampsia in RUPP rats during pregnancy with AT1-AA inhibition, it is not known if AT1-AA inhibition during pregnancy improves the mother’s risk of developing cardiovascular and renal complications postpartum. We hypothesized that AT1-AA inhibition administered during pregnancy would improve PP blood pressure, cardiac function, renal function, and cardiac mitochondrial function, in the pre-clinical rat model of preeclampsia.

## Methods

### Animals

All animal experiments were performed in accordance with the National Institutes of Health guidelines for the use and care of animals. Animal protocols were approved by the Institutional Animal Care and Use Committee (IACUC) at the University of Mississippi Medical Center. Pregnant Sprague Dawley (SD) rats were purchased from Envigo (Indianapolis, IN) and housed in a temperature-controlled room (75° F) with 12-h light–dark cycles per day and with food and water provided ad libitum.

### Postpartum reduced uterine perfusion pressure (RUPP) model and n7AAc infusion

Pregnant SD rats were randomly divided into three groups during pregnancy: Normal pregnant (NP; *n* = 16), reduced uterine perfusion pressure (RUPP; *n* = 15), and RUPP + AT1-AA inhibitory peptide (‘n7AAc’; *n* = 16). On gestational day (GD) 14, RUPP surgery was performed as previously described [40]. This model reduces blood flow to the utero-placental unit by ~ 40% in pregnant rats [40]. One group of RUPP rats randomly received a mini-osmotic pump placed intraperitoneal (IP) to deliver the capped AT1-AA inhibitory peptide (‘n7AAc’; Thermo Fisher Scientific, Waltham, MA) at a dose of 144 μg/day at day 14 of gestation. This dose is based on previous studies performed in our laboratory that demonstrate that AT1-AA inhibition prevents the rise in maternal blood pressure and several pathophysiological factors associated with preeclampsia in RUPP rats, such as the decrease in NO bioavailability, and rise in systemic oxidative stress, pro-antigenic factors, and inflammation [30, 41]. The ‘n7AAc’ was given from gestational day 14 (mid-pregnancy) to postpartum day 6 or 7, depending on when the rats gave birth, which was either on day 21 or 22. Thus the minipump was infused for a total of 14 days, which was 6–7 days during the end of pregnancy and 6–7-day postpartum. ‘n7AAc’ blocks the binding of AT1-AA chronotropic activity [32, 33, 39]. The capped peptide does not bind or alter the function or activity of the AT1 receptor, but specifically binds circulating AT1-AAs without affecting ANG II [33]. All rats were allowed to give birth and nurse their pups. At 10-week postpartum, systemic, cardiac, and renal measurements were made on the dams; and kidneys and the heart was collected for molecular and mitochondrial assays.

### Systemic measurements–blood pressure measurement

At 10-week postpartum, a PE50 catheter was implanted in the right carotid artery and tunneled out of the back of the neck to measure blood pressure. Mean arterial pressure (MAP) was measured with rats in a restrainer cage. Measurements were taken over 30 min after a 30 min equilibration period. Afterwards, animals were sacrificed and blood and tissue collected. Body weight, heart weight, and kidney weight were recorded. Blood plasma and serum were collected and tissue samples stored at − 80 °C for further use [30, 41].

### Systemic measurements–plasma NOx and antioxidant capacity

Plasma nitrate and nitrite was measured using the Nitrate/Nitrite Colorimetric Assay Kit (Cayman Chemical, Ann Arbor, MI) according to the manufacturer’s instructions, as previously described [39]. Plasma total antioxidant capacity was measured using the Antioxidant Assay Kit (709001, Cayman Chemical, Ann Harbor, MI), according to the manufacturer's instruction as previously described [42]. In summary, this assay measures the ability of all aqueous and lipid-soluble antioxidants to inhibit the oxidation of 2,2′-azino-di-(3-ethylbenzthiazoline sulfonate) (ABTS) to ABTS^+^ by metmyoglobin. The total antioxidant capacity is quantified as millimolar Trolox equivalents (mM Trolox) at the 750 nm wavelength.

### Cardiac function–echocardiography

All measurements and assessments were performed using the Visual Sonics Echo system (Vevo 3100, VisualSonics, Inc., Toronto, Canada) and the 15–30 MHz (MX250) linear transducer (VisualSonics). Animals were placed in the echocardiography room for at least 30 min before examination. Rats were maintained unconscious using 2% isoflurane and placed in a supine position on a heating platform. The chest was shaved and ultrasonic gel applied to the thoracic area to allow maximal visibility of the heart chambers. The ultrasonic probe was placed on the chest along the long-axis of the left ventricle and adjusted to obtain clear two-dimensional B-mode and M-mode parasternal long axis images. Five minutes was allowed for each animal to stabilize in that position before acquiring any measurements. Heart rate was maintained constant throughout the procedure (350–400 beats/min). Note that only a subset of rats in the study were used to measure cardiac function. The number of rats used for each experiment are presented in the figure legend.

### Cardiac mitochondria isolation, respiration, and membrane potential

Intact mitochondria were isolated from excised rat hearts by differential centrifugation [10, 43]. In brief, rat hearts were isolated and quickly washed in ice-cold MSM buffer (220 mM mannitol, 70 mM sucrose, 5 mM Mops, pH 7.4). Hearts were covered in ice-cold MSM buffer supplemented with 1 mg/ml bacterial proteinase type XXIV (Sigma) and rapidly minced with a razor blade on a cold and clean plastic surface. The minced tissue was added to ice-cold MSM buffer, supplemented with 2 mM EDTA and 0.2% fatty acid-free BSA, phenylmethylsulfonyl fluoride (PMSF) was added to 0.1 mM, and the tissue was homogenized on ice with a glass homogenizer and a loosely-fitting Teflon pestle using three to four hand driven strokes. The homogenate was centrifuged at 300×*g* for 10 min at 4 °C. The supernatant was centrifuged at 3000×*g* for 10 min at 4 °C, the supernatant was discarded and the pellet containing the mitochondria was resuspended by pipetting with cold MSM buffer with EGTA/BSA, and centrifuged again at 3000×*g*. The final mitochondrial pellet was resuspended in a minimal volume of MSM buffer with EGTA/BSA and the protein concentration was determined using the DCA protein assay (Bio-Rad).

Immediately after isolation, mitochondria were used to measure respiration and membrane potential simultaneously with an Oroboros FluoRespirometer (Oroboros Instruments). The reaction mixture includes 2.1 mL of respiration buffer (100 mM KCl, 5 mM KPi, 1 mM EGTA, 1 mg/ml BSA, 50 mM MOPS, pH 7.4) and 2 μM safranin O (described below). Mitochondria (100–200 μg in 30 µL) were added immediately after oxygen signal stabilization to record respiration (O_2_ consumption) driven by endogenous substrates in the isolated mitochondria. State 2 respiration was initiated by injecting glutamate (10 mM) and malate (2 mM) into the chamber. More rapid State 3 respiration was initiated by adding ADP (5 mM), which allows proton flow back across the inner mitochondrial membrane through ATP synthase. Then oligomycin (2.5 µM) was added, to inhibit proton flow through ATP synthesis, yielding the slower rate of State 4 respiration. Rotenone (0.5 µM) and antimycin A (2.5 µM) were injected to inhibit electron transfer to O_2_ that is specific to oxidative phosphorylation. The resulting slow rates of O_2_ consumption, due to processes other than oxidative phosphorylation, were subtracted from other rates of O_2_ consumption. Rates of respiration are expressed as nmol e-/min/mg mitochondrial protein.

Mitochondrial membrane potential was measured by using the membrane-permeable cationic, fluorescent dye safranin O (*Ex*/*Em* = 485 nm/586 nm) as previously described [16, 21]. Safranin is taken into the matrix in proportion to the density of negative charge on the matrix surface of the inner mitochondrial membrane. Since the crowding of safranin in the matrix causes quenching of its fluorescence, safranin fluorescence is inversely proportional to the magnitude of the membrane potential [43]. The fluorescence signal of the Oroboros FluoRespirometer is calibrated using known concentrations of safranin up to 2 μM maximum. Hence, the fluorescence readout of the Oroboros software is in units of 0–2 µM safranin, where the concentration of safranin is that of the fluorescent population on the outside of the mitochondria. This is easily converted to percent uptake of the total amount of safranin in the reaction; therefore, our relative measure of the magnitude of mitochondrial membrane potential is “percent safranin uptake”. The maximum percent safranin uptake value, reported here, is taken at the point, where safranin fluorescence quenching is greatest. The magnitude of the membrane potential is maximum during State 2 and State 4 respiration, it declines slightly during State 3 respiration, and the membrane potential is completely lost (safranin fluorescence returns to maximum) upon the addition of an uncoupler, such as FCCP.

Uncontrolled complex IV activity was measured as the rate of oxygen consumption catalyzed by complex IV in broken, KCl-washed rat heart mitochondria as described previously [44] with some modifications. Electrons were provided to complex IV in 10 μg broken mitochondria by horse heart cytochrome *c* (20 μM), which was kept reduced by ascorbate (3 mM) and *N*,*N*,*N*′,*N*′-tetramethyl-*p*-phenylenediamine (TMPD; 0.3 mM). The reaction mixture also contained 50 mM Tris (pH 7.4), 8 mM KCl, 1 mM EDTA, 2 μg/ml catalase, 5 μM antimycin A, at 25 °C. Inhibition of complex IV activity upon the addition of 25 mM ZnSO_4_ and 5 mM MgCl_2_ allowed measurement of the slow, non-enzymatic consumption of O_2_ by ascorbate/TMPD, which was subtracted. Complex IV activity is reported as nmol e-/min/mg protein.

### Renal function

Renal function was determined by glomerular filtration rate (GFR) using FITC-sinistrin as previously described [39, 45]. Briefly, 1–2 days before rats were 10-week postpartum a catheter was inserted into the jugular vein to infuse FITC-sinistrin under isoflurane anesthesia. At 10 weeks, postpartum rats were anesthetized and hair on the upper back below the ears was removed to reduce interference. For determining GFR, a miniaturized device (NIC-Kidney, Mannheim Pharma & Diagnostics, Mannheim, Germany) composed of 2-light-emmiting diodes that transcutaneously excite and measure FITC-sinistrin was used and baseline fluorescence collected for 10–15 min, followed by a bolus injection of FITC-sinistrin (3 mg/100 g body weight in 0.2 mL 0.9% irrigation saline). Continuous fluorescence was measured for 2 h and clearance curves analyzed using the MPD Lab Ver 1.0RC3 software. The half-life (*t*_1/2_) for the clearance of FITC-sinistrin was determined 45 min post-injection using a one-compartment model. The t_1/2_ value was converted to GFR (mL/min/100 g body weight) using the following semi-empirical equation developed and validated by the manufacturer: GFR = 31.26 [mL/100 g body weight]/*t*_1/2_ [min] [45]. Due to the invasiveness and surgical stress of determining renal function measurements in rats, only a subset of the rats in the study were used to measure GFR. The number of rats used for each experiment are presented in the figure legend.

### Plasma creatinine

Plasma creatinine was measured using the LabAsay Creatinine kit (Jaffe method; Wako Pure Chemical Industries, Ltd., Osaka, Japan) according to the manufacturer’s instructions and as previously published [39]. The standards ranged from 1.25 to 10 mg/dl. The amount of plasma creatinine was measured calorimetrically and expressed as mg/dL.

### Proteinuria

Proteinuria was measured by a BCA protein assay from Bio-Rad using bovine albumin as standard. Briefly, rat urine was collected for 24 h in metabolic cages at 10-week postpartum and diluted 1:10 with 1 × phosphate buffered saline (PBS). Diluted samples were pipetted into duplicates wells and measured by the spectrophotometer at 540 nm. Proteinuria was expressed as mg/day.

### Urine nephrin excretion

Urine nephrin excretion was measured by the Exocell rat nephrin ELISA kit (kit #:1019; Exocell Inc.) according to the manufacturer’s instructions. Urine nephrin excretion was expressed as µg/mL. The urine used in this assay collected for 24 h in metabolic cages at 10-week postpartum.

### Western blot analysis

Cardiac tissue was homogenized in a RIPA buffer with protease and phosphatase inhibitors using the Fisher Scientific PowerGen 125 electric homogenizer at a low speed. Lysates were separated into a membrane and soluble fraction by centrifugation (10,000×*g*) for 30 min. Samples containing 100 µg of protein from the soluble fraction were loaded on 4–20% precast Criterion gels (Bio-Rad, cat # 5671093). Separated proteins were transferred to nitrocellulose membranes using a trans-blot turbo apparatus (Bio-Rad), which were blocked for 1 h at room temperature in blocking buffer (LI-COR Biosciences, Lincoln, NE) diluted 1:1 with PBS. Membranes were incubated overnight at 4 °C with electron transport chain primary antibody total OXPHOS (1:250; Abcam, Cambridge. MA; cat # ab110413). This cocktail of antibodies recognizes complex I–V proteins of the electron transport chain. Membranes were washed 3× in PBS with 0.1% Tween-20) and incubated with IRDeye700-conjugated anti-mouse IgG (1:5000, LI-COR Biosciences, cat # 928-68070) for 1 h and scanned using the LI-COR Odyssey Infrared Imaging System.

### Statistical analysis

Mean values ± SEM for n rats or independent observations are presented. The significance of differences among mean values was analyzed by a one-way ANOVA with Bonferoni’s post hoc test. Two means were compared using an unpaired *t* test. All statistical analysis was performed with Prism 8 (GraphPad Software, La Jolla, CA). *p* < 0.05 was considered statistically significant**.**

## Results

**Fig. 1:**
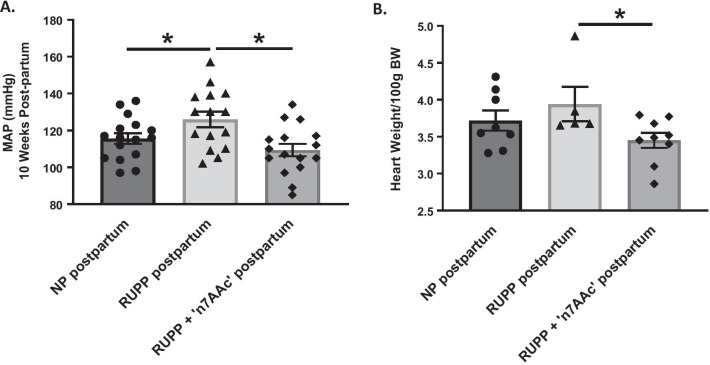
Postpartum effects of AT1-AA blockade in preeclampsia on blood pressure and heart weight. **A** blood pressure was measured by carotid catheter at 10-week postpartum in rats that underwent normal pregnancy (NP), RUPP-induced preeclampsia, or the RUPP procedure and treatment with ‘n7AAc’ to inhibit AT1 autoantibodies (AT1-AA). **B** hearts were extracted at 10-week postpartum and normalized to body weights (BW). Statistical comparisons between normal pregnant (NP) postpartum, RUPP postpartum, and RUPP + AT1-AA inhibition (‘n7AAc’) postpartum was analyzed by a one-way ANOVA with Bonferoni’s post hoc test. *p ≤ 0.05 was considered statistically significant (*n* = 4–16)

At 10-week postpartum, MAP was elevated in RUPP vs. NP rats (126 ± 4 vs. 116 ± 3 mmHg, *p* < 0.05) (Fig. 1A). RUPP + 7AA displayed a 17 mmHg drop in MAP compared to RUPP rats postpartum (109 ± 3 vs. 126 ± 4 mmHg, *p* < 0.05) (Fig. 1A), suggesting that AT1-AA inhibition during pregnancy prevents the increase in MAP at 10-week postpartum in RUPP rats and restores MAP to NP levels (Fig. 1A). Heart weights in RUPP rats postpartum vs. NP postpartum were not significantly different (*p* = 0.39, ns). However, RUPP + 7AA vs. RUPP rats heart weights were significantly lower (3.45 ± 0.10 vs. 3.94 ± 0.23 g/100 g BW, *p* < 0.05) (Fig. 1B and Table 2).

There was no difference in body and kidney weights between 10-week postpartum rats (Table 1). GFR, a marker for kidney function, which was determined by sinistrin infusion, was the same in NP postpartum rats, RUPP rats, and RUPP + 7AA rats (Table 1). Furthermore, there was no change in plasma creatinine, proteinuria, and urine nephrin excretion, suggesting no drastic renal injury or function of RUPP rats postpartum (Table 1).

**Table 1:** Ten-week postpartum renal function outcomes

	NP post-partum	RUPP post-partum	RUPP + ‘n7AAc’ post-partum
Kidney weight (g)/1000 g BW	6.06 ± 0.12	6.13 ± 0.18	5.96 ± 0.17
Glomerular filtration rate (GFR) (mL/min)	1.19 ± 0.25	0.86 ± 0.07	1.00 ± 0.03
Plasma creatinine (mg/dL)	0.52 ± 0.02	0.51 ± 0.02	0.56 ± 0.04
Proteinuria (mg/day)	167.1 ± 11.6	161.5 ± 52.4	157.2 ± 20.4
Urine nephrine (μg/mL)	6.37 ± 1.56	6.10 ± 0.08	7.63 ± 0.91

Ten weeks postpartum renal function outcomes for normal pregnant (NP) postpartum, RUPP postpartum, and RUPP + AT1-AA inhibition (‘n7AAc’) postpartum rats. All statistical analyzes were performed by a one-way ANOVA with Bonferoni’s post hoc test

There was a decrease in stroke volume (SV) as measured by echocardiography in RUPP vs. NP rats postpartum (Table 2). Moreover, there was a tendency toward lower cardiac output (SV × heart rate) in RUPP rats. However, ejection fraction (SV/end-diastolic volume × 100) was not different in the RUPP group, suggesting a reduction in end-diastolic volume. Together these findings are consistent with modest concentric hypertrophy in the RUPP rats with possible diastolic dysfunction. No changes in cardiac output, ejection fraction, or fractional shortening was found between NP and RUPP + 7AA rats postpartum (Table 2).

**Table 2:** Ten-week postpartum cardiac function outcomes

	NP post-partum	RUPP post-partum	RUPP + ‘n7AAc’ post-partum
Body weight (g)	254 ± 4	259 ± 3	253 ± 2
Heart weight/100 g BW (g)	3.72 ± 0.14	3.94 ± 0.23	3.45 ± 0.10^+^
Strike volume (mL)	188 ± 7	167 ± 5*	184 ± 12
Cardiac output (mL/min)	56 ± 2	52 ± 2	59 ± 3
Ejection fraction	76 ± 2	77 ± 2	76 ± 2
Fractional shortening	45 ± 2	47 ± 2	47 ± 2

Ten weeks postpartum cardiac function outcomes for normal pregnant (NP) postpartum, RUPP postpartum, and RUPP + AT1-AA inhibition (‘n7AAc’) postpartum rats. All statistical analyzes were performed by a one-way ANOVA with Bonferoni’s post hoc test. *p ≤ 0.05 vs. NP and ^+^*p* ≤ 0.05 vs. RUPP for *n* ≥ 6 rats

*p < 0.05 vs. NP postpartum; ^+^*p* < 0.05 vs. RUPP postpartum rats

**Fig. 2:**
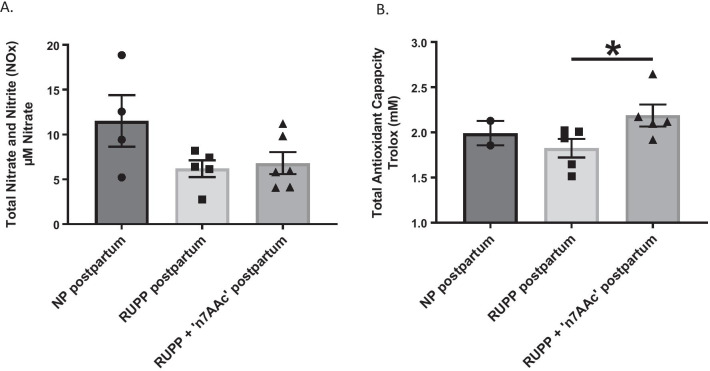
Postpartum effects of AT1-AA blockade in preeclampsia on plasma nitric oxide (NO) and antioxidant capacity. **A** plasma NO metabolites nitrate and nitrite were measured using a commercially available kit. **B** plasma total antioxidant capacity was measured as the ability of all aqueous and lipid-soluble antioxidants to inhibit the oxidation of ABTS to ABTS + by metmyoglobin. The total antioxidant capacity was quantified as millimolar Trolox equivalents. All statistical comparisons between normal pregnant (NP) postpartum, RUPP postpartum, and RUPP + AT1-AA inhibition (‘n7AAc’) postpartum was analyzed by a one-way ANOVA with Bonferoni’s post hoc test. *p ≤ 0.05 was considered statistically significant (*n* = 2–6)

There was a modest trend towards a decrease in circulating nitrate and nitrite concentrations for RUPP vs. NP rats postpartum (6.19 ± 0.9 vs. 11.5 ± 2.9 µM nitrate, *p* = 0.09) (Fig. 2A), suggesting a decrease in NO bioavailability for RUPP rats. The average amount of circulating plasma total antioxidant capacity in RUPP and NP rats postpartum was the same (Fig. 2B). However, there was significant increase in total antioxidant capacity for RUPP + 7AA vs. RUPP rats postpartum, demonstrating that antioxidant capacity increases with AT1-AA inhibition during pregnancy (2.19 ± 0.12 vs. 1.83 ± 0.10 mM Trolox, *p* < 0.05) (Fig. 2B).

**Fig. 3:**
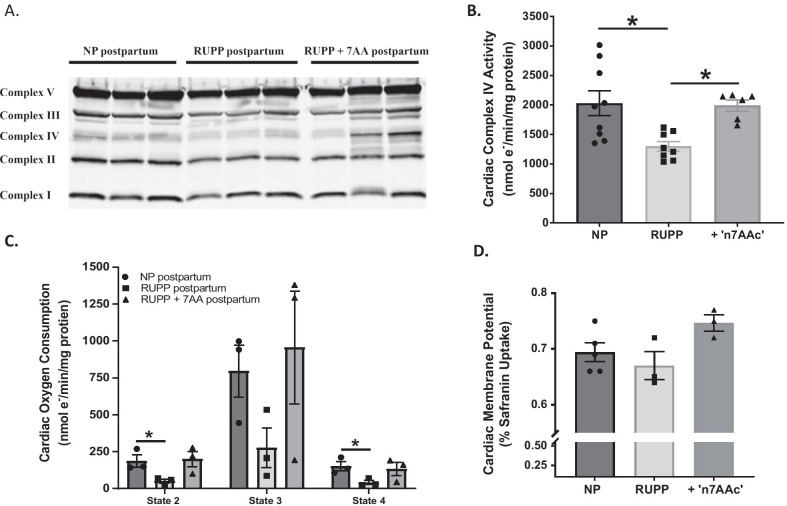
Postpartum effects of AT1-AA blockade in preeclampsia on cardiac mitochondrial complexes and function. Mitochondria were isolated from hearts 10-week postpartum of rats that underwent normal pregnancy (NP), RUPP-induced preeclampsia, or the RUPP procedure and treatment with ‘n7AAc’ to inhibit AT1 autoantibodies (AT1-AA). **A** representative Western blot for respiratory complexes showing a reduction in levels of complex IV with RUPP-induced preeclampsia and recovery with AT1-AA blockade. **B** complex IV activity is reduced postpartum in mitochondria from RUPP rats compared to NP or RUPP + ‘n7AAc’. *p ≤ 0.05 was considered statistically significant (*n* = 6–9). **C** states 2, 3, and 4 respiration showing an overall reduction in mitochondria of RUPP rats postpartum compared to NP or RUPP + ‘n7AAc’. *p ≤ 0.05 was considered statistically significant (*n* = 3) **D** mitochondrial membrane potential with state 2 respiration measured by safranin uptake. *p ≤ 0.05 was considered statistically significant (*n* = 3–5). Note that statistical comparisons between normal pregnant (NP) postpartum, RUPP postpartum, and RUPP + AT1-AA inhibition (‘n7AAc’) postpartum was analyzed by a one-way ANOVA with Bonferoni’s post hoc test for figures **B**–**D**

Cardiac cytochrome oxidase (complex IV) protein was decreased in RUPP vs. NP rats postpartum (86 ± 6 vs. 100 ± 0.01% fold of complex IV/Ponceau/NP postpartum, *p* < 0.05) (Fig. 3A and Additional file 1). Complex IV levels were restored in RUPP + 7AA rats postpartum (102 ± 8% fold of complex IV/Ponceau/NP postpartum) (Fig. 3A and Additional file 1). No other mitochondria electron transport chain proteins (complexes I, II, III, V) were changed among the groups (Fig. 3A). Moreover, RUPP hearts contained less complex IV activity, normalized to mitochondrial protein, which was restored in RUPP + 7AA hearts (Fig. 3B). Cardiac mitochondria from RUPP rats had lower rates of state 2, 3 and 4 respiration in comparison to NP and RUPP + 7AA rat cardiac mitochondria (Fig. 3C). There was no difference in mitochondrial membrane potential during state 2 respiration among the groups (Fig. 3D), indicating no proton leak.

## Discussion

Women with hypertensive pregnancies have an increased risk of developing CVD, hypertension, and renal disease postpartum [1]. This paper is the first show that RUPP rats maintain increased blood pressure associated with reduced cardiac mitochondrial dysfunction 10-week postpartum. Importantly, we also show that AT1-AA inhibition during pregnancy normalized postpartum hypertension and cardiac mitochondrial function, thus indicating the importance of the AT1-AA to contribute to the pathophysiology of PE in the mother during pregnancy, as previously published [39], as well as her cardiovascular health later in life. Furthermore, this study suggests that improvements in antioxidant capacity by AT1-AA blockade in postpartum hypertensive rats is beneficial to the mother’s postpartum cardiovascular health. The observed improvements with AT1-AA inhibition in mitochondrial respiration and complex IV electron transport chain in the preclinical rat model of preeclampsia, suggests that improvements in cardiac mitochondrial function could prevent postpartum CVD and hypertension.

AT1-AAs are elevated during preeclampsia and in the RUPP preclinical rat model of preeclampsia [1, 4, 8, 9, 30–33] and are elevated in women up to 2 years after delivery [46]. The presence of AT1-AAs postpartum may be contributing to the hypertension observed in RUPP rats at 10-week postpartum; however, the importance of our study shows that blockade of AT1-AA during pregnancy prevents hypertension and cardiac hypertrophy and mitochondrial dysfunction that occurs postpartum in the RUPP rat. Although the AT1-AA was not measured in PP RUPP rats, treatment with the 7AA during pregnancy prevented cardiac pathology observed at 10-week PP following a placental ischemic pregnancy.

Pregnant rats administered AT1-AAs exhibit increased in blood pressure, oxidative stress, inflammation, and decrease in NO bioavailability [30, 37, 47–51]. Likewise, AT1-AAs in pregnant rats also caused placental ischemia [52, 53], which is believed to be the initial and major contributing factor to the development of preeclampsia [54]. Not only does AT1-AA cause vasoconstriction of the placental vasculature, but also the renal afferent arterioles causing an increase in renal artery resistance index and renal vascular resistance in pregnant rats [30, 37]. Studies done by our lab have shown that AT1-AA increases ANG II-induced renal vascular resistance and decreases GFR, an index of kidney function, in pregnant rats [30]. Data from this previous study propose that AT1-AAs act to increase ANG II sensitivity synergistically as is observed in women with preeclampsia [4, 33]. It is important to note that the increase in renal vascular resistance and decrease in GFR observed in the previous study was correlated with a threefold systemic increase in plasma isoprostane levels, a marker for oxidative stress [30]. AT1-AAs increase placental oxidative stress [37], partly due to activation of NADPH oxidase, which produces superoxide, a potent reactive oxygen species that is elevated in preeclamptic patients and can cause vascular dysfunction, poor invasion of trophoblast cells for placentation, placental ischemia, and a decrease in NO bioavailability [55–57]. These data demonstrate that oxidative stress via AT1-AA alone or in the presence of ANG II, greatly contributes to the pathophysiology of PE [30]. Moreover, inhibition of AT1-AA activity with the ‘n7AAc’ inhibitory peptide ameliorated hypertension, mitochondria oxidative stress, NO bioavailability, and other markers of preeclampsia [39]. Thus, AT1-AA inhibition could be a good therapy for women during preeclampsia. Moreover, in this study we demonstrate the potential health benefits AT1-AA inhibition during preeclampsia can have on the postpartum period. Of note, ‘n7AAc’ did not improve NO availability although the antioxidant capacity was increased in that group. Superoxide can limit NO bioavailability by two means; one is by combining with NO to produce peroxynitrite [58, 59] or by uncoupling the Nitric Oxide Synthases (NOS) to produce superoxide instead of NO [60, 61]. Despite the increase in antioxidants, there can still be superoxide present, which will limit NO bioavailability. In addition, the antioxidant assay considers all different types of antioxidants, which would for instance include catalase and not just superoxide dismutase. More experiments are need to identify the exact changes of antioxidants in these studies.

Within the mitochondria electron transport chain, complex IV or cytochrome C oxidase is the ultimate enzyme in this cascade of that passes electrons to oxygen to produce water and aids in the generation of the proton gradient for cellular respiration and energy supply. Several studies have shown that complex IV activity is decreased in the preeclamptic placenta [62–64]. Mitochondria dysfunction and reactive oxygen species are also elevated in preeclamptic women and animal models of preeclampsia [8, 10, 65–72]. Previous studies show that the preclinical RUPP model displays placental and renal mitochondrial oxidative stress that is decreased by AT1-AA inhibition during pregnancy [8, 10]. Placental mitochondrial respiration and electron transport chain protein and activity is also reduced in RUPP rats vs. NP rats during pregnancy [10]. RUPP rats treated with mitochondrial specific antioxidants improved not only mitochondrial function and oxidative stress, but also hypertension and other pathologies of preeclampsia [10], signifying that mitochondrial dysfunction and oxidative stress contribute to the pathology of preeclampsia. [73]. In this study we found that complex IV mitochondrial protein abundance and activity in the heart was decreased in RUPP rats postpartum. Interestingly, Complex IV protein abundance and activity was also decreased during pregnancy in the placenta of RUPP vs. NP rats [10]. Importantly, cardiac Complex IV expression and function were normalized postpartum with AT1-AA inhibition during pregnancy, suggestive of improved mitochondrial function. Additional studies are needed to establish the mechanism of action by which AT1-AA causes long term changes in the heart and how these are reversed by ‘n7AAc’. As with in utero stress on the fetus, epigenetic programing may be involved downstream of AT1 receptor activation by AT1-AA in the maternal heart [74], with ‘n7AAc’ attenuating this activation by serving as a “decoy receptor”.

Similar to the trend for postpartum preeclamptic women to develop CVD and hypertension, postpartum RUPP rats, had a ~ 11 mmHg increase in MAP which was attenuated by AT1-AA inhibition during the pregnancy. Decreased NO bioavailability and increased oxidative stress contribute to development of hypertension and endothelial dysfunction. We did not observe any differences among the groups in circulating total nitrate and nitrite, metabolites of NO; however, there was a decrease in total antioxidant capacity in RUPP rats postpartum in which was reversed in RUPP rats treated with AT1-AA inhibition during pregnancy.

Preeclamptic women 4-month to 20-year postpartum are reported to have a decrease in renal function [1, 18, 24–26, 28]. The decrease is variable with many studies showing a small but not statistically significant decrease in GFR like our study [1, 26]. Most clinical studies do not show a change in plasma creatinine levels or proteinuria in postpartum preeclamptic women [1, 26]. Similar to some of these clinical studies, there were no significant changes were observed with serum creatinine levels and estimated glomerular filtration rate in our study [23]. In addition, there were no changes in postpartum body and kidney weights among the groups, nor in markers of renal injury, such as plasma creatinine and proteinuria. Podocyturia is present in preeclamptic women during pregnancy and 6–8-week postpartum [75]. Nephrin excretion is a marker of podocyte injury which is considered an index of renal injury. However, we saw no difference in nephrin excretion, supporting the conclusion that renal injury is not evident in the postpartum RUPP rat.

Several limitations to our study should be acknowledged. First, we did not measure AT1-AA postpartum levels, although others have and show it present up to 2-year postpartum in humans [46]. Second, more experiments are warranted to establish whether the improved post-partum outcomes are due to AT1-AA sequestering or the lack of increased blood pressure during pregnancy as the mechanism of ‘n7AAc’ actions to facilitate the changes we observed at 10-week postpartum. Third, we decided to conduct our studies at 10 weeks, because that is when we first observe physiological changes. Future experiments will observe more timepoints, especially after the 10-week period, for which we hypothesize to see more drastic changes in blood pressure, cardiac dysfunction, and cardiac mitochondria function. Four, we examined NOX and antioxidant levels, which are both indirect measures of NO bioavailability, a marker of endothelial function. Future experiments will measure more markers of endothelial damage, such as ex vivo vascular studies and endothelin 1, which is elevated in PE. Finally, the clips used in the RUPP model were not removed after pregnancy in order not to elicit additional stress. After deliver of the pups, the clips around the left and right uterine arcade are no longer functional, while the distal clip above the iliac bifurcation does not block blood flow by 100% or cause any harm or known side effects to rats after pregnancy.

## Perspectives and significance

In summary, this study shows for the first time that AT1-AA inhibition during a hypertensive pregnancy in rodents, improves postpartum blood pressure and cardiac mitochondrial function. We believe that the mechanisms of improved outcomes could be attributed to the increase in systemic antioxidant capacity and normalized cardiac mitochondrial function. This study indicates the importance of AT1-AA inhibition during pregnancy to not only benefit the preeclamptic women during pregnancy, but also to prevent her risk of developing hypertension and CVD later in life.

## Supplementary Information

**Additional file 1: Figure 1.** Quantification of the immunoblots for complex protein levels. Statistical comparisons between normal pregnant (NP) postpartum and RUPP postpartum was analyzed by student *t* test *p ≤ 0.05.

## Data Availability

Please contact author for data requests.

## Abbreviations

PE: Preeclampsia
CVD: Cardiovascular disease
AT1-AA: Angiotensin II type I receptor agonistic autoantibodies
PP: Postpartum
RUPP: Reduced Uterine Perfusion Pressure Model
‘n7AAc’: Capped specific 7 amino acid peptide binding sequence
NP: Normal pregnant
MAP: Mean arterial pressure
GFR: Glomerular filtration rate
SD: Sprague Dawley rat
