# Supplementary figures and images for: Angiotensin II type 1 receptor agonistic autoantibody blockade improves postpartum hypertension and cardiac mitochondrial function in rat model of preeclampsia
Source: Biol Sex Differ. 2021 Nov 2;12:58. doi: 10.1186/s13293-021-00396-x (PMC8562001; doi:10.1186/s13293-021-00396-x)

## Slide 1
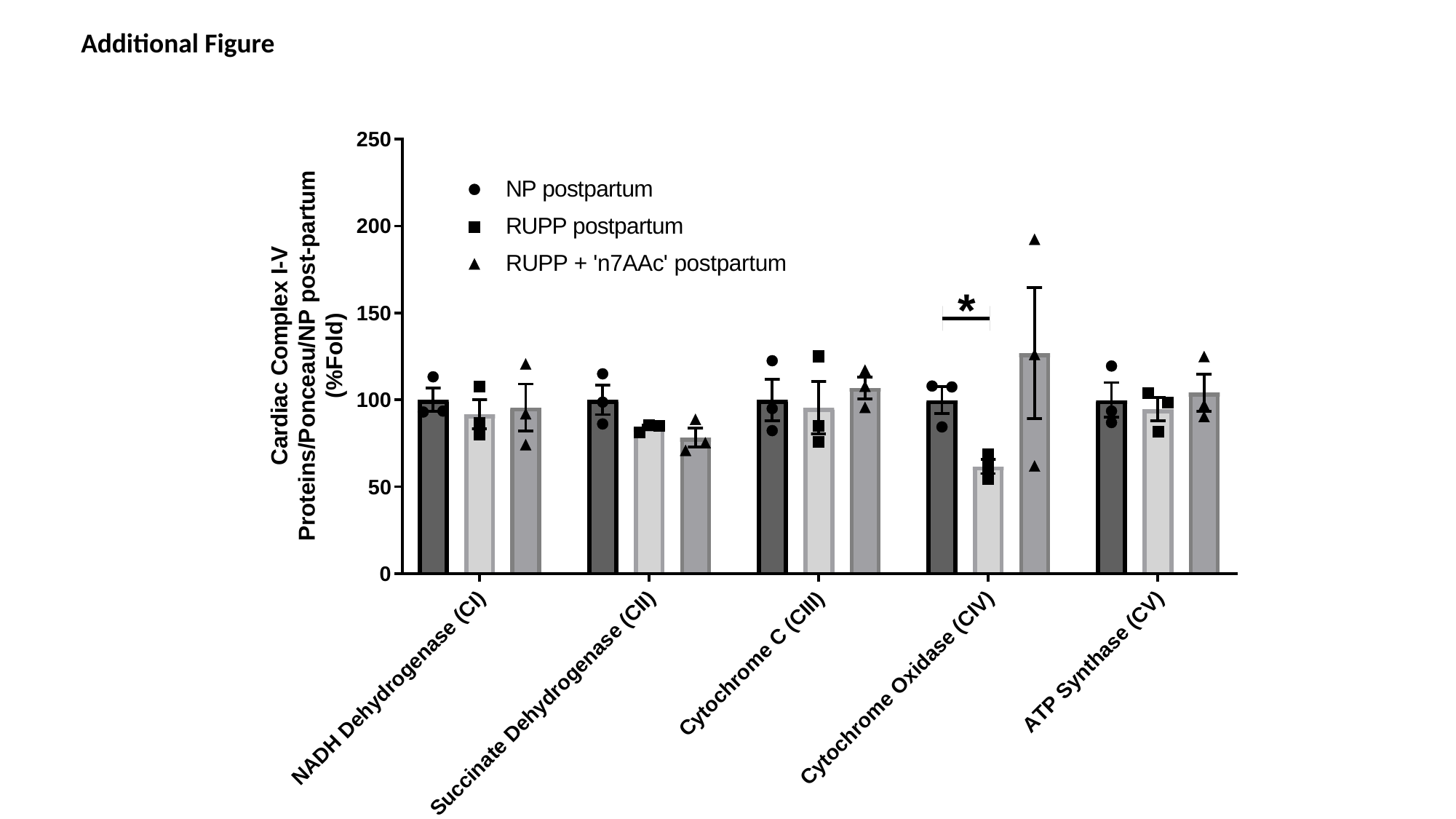

Additional Figure

Supplement: Supplementary file 1 — Additional file 1: Figure 1. Quantification of the immunoblots for complex protein levels. Statistical comparisons between normal pregnant (NP) postpartum and RUPP postpartum was analyzed by student t test *p ≤ 0.05. [file 13293_2021_396_MOESM1_ESM.pptx]
